# Recovery in pupillometric non-visual functions following chiasmal decompression in pituitary adenoma

**DOI:** 10.1093/braincomms/fcag233

**Published:** 2026-06-19

**Authors:** Daniella Mahfoud, Jensen Ang, Beng-Ti Ang, Monisha Esther Nongpiur, Dan Milea, Raymond P Najjar

**Affiliations:** Eye N’ Brain Research Group, Department of Ophthalmology, Yong Loo Lin School of Medicine, National University of Singapore, Singapore 119228, Singapore; Visual Neurosciences Research Group, Singapore Eye Research Institute, Singapore 169856, Singapore; Department of Neurosurgery, National Neuroscience Institute (Singapore General Hospital Campus), Singapore 168581, Singapore; SingHealth Duke-NUS Neuroscience Academic Clinical Programme, Duke-National University of Singapore Medical School, Singapore 169857, Singapore; Department of Neurosurgery, National Neuroscience Institute (Singapore General Hospital Campus), Singapore 168581, Singapore; Neuro-Oncology Research Laboratory, Department of Research, National Neuroscience Institute, Singapore 308433, Singapore; Visual Neurosciences Research Group, Singapore Eye Research Institute, Singapore 169856, Singapore; Departments of Neuro-ophthalmology and Glaucoma, Singapore National Eye Center, Singapore 168751, Singapore; Ophthalmology and Visual Sciences ACP, Duke-NUS Medical School, Singapore 169857, Singapore; Visual Neurosciences Research Group, Singapore Eye Research Institute, Singapore 169856, Singapore; Departments of Neuro-ophthalmology and Glaucoma, Singapore National Eye Center, Singapore 168751, Singapore; Ophthalmology and Visual Sciences ACP, Duke-NUS Medical School, Singapore 169857, Singapore; Department of Ophthalmology, Rothschild Foundation Hospital, Paris 75019, France; Eye N’ Brain Research Group, Department of Ophthalmology, Yong Loo Lin School of Medicine, National University of Singapore, Singapore 119228, Singapore; Visual Neurosciences Research Group, Singapore Eye Research Institute, Singapore 169856, Singapore; Ophthalmology and Visual Sciences ACP, Duke-NUS Medical School, Singapore 169857, Singapore; Department of Biomedical Engineering, College of Design and Engineering, National University of Singapore, Singapore 119077, Singapore

**Keywords:** pituitary adenoma, transsphenoidal surgery, pupillometry, optic chiasm, visual recovery

## Abstract

Pituitary adenomas frequently compress visual and non-visual pathways, resulting in visual field deficits and sleep disturbances. While transsphenoidal surgery is often anatomically effective, functional visual and non-visual outcomes remain variable and often unpredictable. This study investigates visual and non-visual (i.e. pupillometric) changes associated with optic nerve compression and decompression and the potential role of chromatic pupillometry as an objective evaluation tool for visual dysfunction in pituitary adenoma. This longitudinal study included 27 patients with pituitary adenoma [median age = 55.4 years (interquartile range = 23.4)] and 41 age-matched controls [53.6 (13.7) years], evaluated pre-operatively (mean duration ± SD: 7.0 ± 5.4 weeks prior to surgery) and post-operatively (12.0 ± 1.5 weeks post-surgery) with handheld chromatic pupillometry, in addition to comprehensive neuro-ophthalmological and neuroimaging examinations. Pupillometric features were analysed for associations with structural changes and visual outcomes. There was a significant reduction in the upward displacement and increase in the thickness of the optic chiasm measured on MRI (*P* *<* 0.001) following transsphenoidal surgery. Pupillary responses improved post-operatively, including increased maximum constriction to both red and blue light, but remained below control levels (*P* *<* 0.05). In patients with full visual field index recovery (*n* *=* 5), pupillometric responses were comparable to controls (*P* *>* 0.05) while patients without recovery exhibited persistent deficits in these metrics. Remarkably, melanopsin-driven post-illumination pupil responses improved significantly, reaching control values post-operatively (*P* *>* 0.05), regardless of visual field index recovery. Structural recovery correlated with improved visual field index (*ρ* = −0.62, *P* *<* 0.001) and with maximum constriction to red light (*ρ* = −0.47, *P* *=* 0.004). Following transsphenoidal surgery, there was a consistent recovery in pupillary light responses that correlated with clinical structural and functional changes observed in pituitary adenoma patients. Notably, melanopsin-mediated responses normalized post-operatively even in patients without visual field recovery, suggesting that non-visual pathways can recover independently of vision. Handheld chromatic pupillometry is a promising, non-invasive biomarker to track both visual and non-visual outcomes. Future studies integrating circadian and sleep markers are warranted to establish its role as a proxy for systemic non-visual functions in this population.

## Teaser

Mahfoud *et al*. report that quantitative chromatic pupillometry can reveal functional recovery of photoreceptive neural pathways following surgical decompression of the optic chiasm in patients with pituitary adenoma. Their findings show that pupil responses can improve independently of vision, highlighting pupillometry as a non-invasive marker of brain function restoration.

## Graphical Abstract

**Graphical Abstract:**
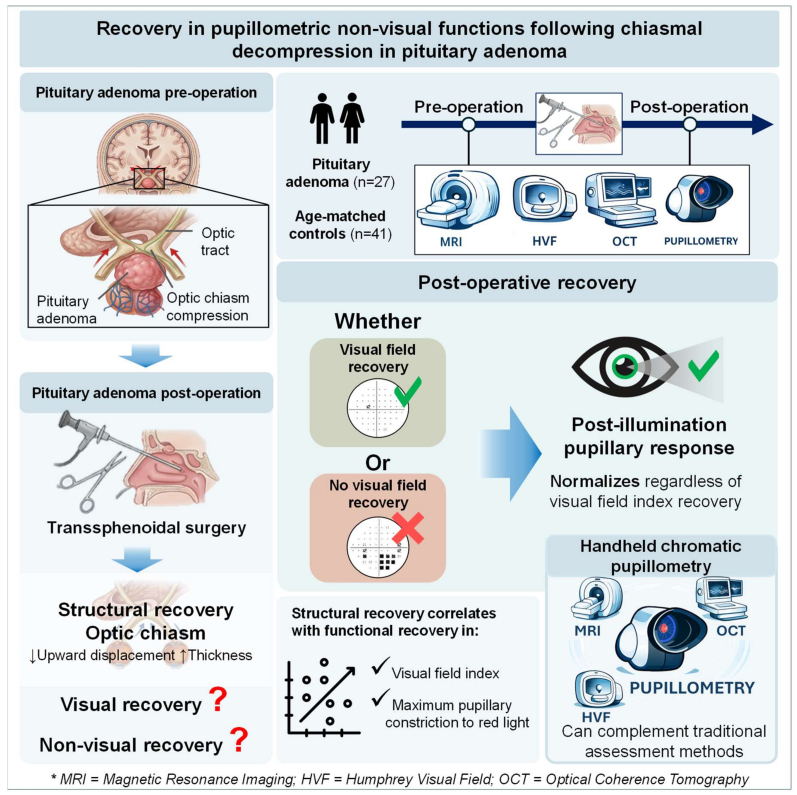

## Introduction

Pituitary adenomas (PAs) are common intracranial tumours that can compress the visual pathways, causing visual impairment in 30–70% of cases^1^ and significantly affecting quality of life. Although transsphenoidal surgery (TSS) is the standard treatment for PAs,^2^ visual recovery following surgery varies widely and is not always predicted by anatomical restoration seen on MRI.^3,4^ Conventional evaluation methods such as automated perimetry, optical coherence tomography (OCT), diffusion-weighted MRI and visual evoked potentials (VEP),^5,6^ are often complex, time-consuming or patient-dependent. While OCT has demonstrated strong diagnostic utility in detecting compressive chiasmopathy and predicting visual recovery based on retinal nerve fibre layer (RNFL) thinning,^1,7^ it primarily reflects structural damage rather than real-time neuro-ophthalmic function. Similarly, MRI delineates chiasmal displacement but lacks predictive power for functional outcomes.^6^ Perimetry relies on patient cooperation,^8^ and VEP, though objective and clinically valuable, is time-intensive, limiting its widespread clinical utility.^9^ These limitations highlight the need for a rapid, objective and accessible method for tracking neuro-ophthalmic function in PA patients.

Quantitative pupillometry has emerged as a promising tool for assessing neuro-ophthalmic function^10,11^ with studies showing improvement in indices like the neurological pupil index following TSS, paralleling visual acuity gains.^12,13^ However, its potential for tracking visual field recovery and chiasmal plasticity remains unexplored. Chromatic pupillometry, which selectively stimulates different photoreceptor pathways, offers added functional insights. Rods and cones mediate transient pupillary constriction, while intrinsically photosensitive retinal ganglion cells (ipRGCs) drive sustained responses, particularly to blue light.^14,15^ These ipRGC-mediated responses form the principal retinal input to the pupillary light reflex (PLR), a well-characterized non-visual response. In this pathway, retinal signals are transmitted from ipRGCs to the olivary pretectal nuclei, which then project bilaterally to the Edinger–Westphal nuclei. Parasympathetic efferents subsequently travel via the oculomotor nerve to the ciliary ganglion and iris sphincter muscle, resulting in pupil constriction.^16^ Owing to its robustness, objectivity and direct dependence on ipRGC signalling, chromatic PLR assessment has become a widely used surrogate marker of non-visual photoreception and retinal–optic nerve integrity in both health and neuro-ophthalmic disease.^15,17-21^ Importantly, ipRGC-driven responses also underpin a wide range of other non-visual functions, including circadian photoentrainment, melatonin suppression and sleep regulation.^22^ These effects are mediated primarily through direct projections via the retinohypothalamic tract to the suprachiasmatic nucleus (SCN), the central pacemaker coordinating circadian rhythms and downstream physiological processes.^23^ In addition to the SCN, ipRGCs project to multiple subcortical non-visual brain regions implicated in alertness, mood, emotional processing and cognitive state, highlighting their broad influence on brain function beyond image formation.^24^

Evidence linking optic nerve disorders to sleep and circadian disruption remains limited, with most data derived from glaucoma populations or mitochondrial optic neuropathies.^25-29^ Therefore, chromatic pupillometry may offer insights into physiological functions that extend beyond vision, which may also be disrupted in patients with PA.^30^ For instance, the post-illumination pupil response (PIPR), largely driven by melanopsin-expressing ipRGCs, is a robust marker of non-visual light responses^31,32^ and may reveal differential recovery trajectories that are not apparent through conventional ophthalmic tests. Investigating these pathways could expand our understanding of PA surgery outcomes and provide a functional biomarker of visual recovery.

In this study, we used handheld chromatic pupillometry (HCP) to investigate dynamic pupillometric features before and after TSS and their relationships with visual outcomes. We hypothesize that improvements in pupillary responses, such as maximum constriction amplitudes and PIPRs, will parallel structural and functional recovery, providing an objective indicator of both neuro-ophthalmic and non-visual outcomes following surgical decompression.

## Materials and methods

### Patient consent for publication

Written infromed consent was obtained directly from all participants. The study was approved by the SingHealth Centralised Institutional Review Board [CIRB (2018/3233)].

### Participants

This prospective, longitudinal study included a total of 27 patients with PA and 41 healthy controls. Patients were recruited from the Neurosurgery and Neuro-Ophthalmology clinics at Singapore General Hospital (SGH), while healthy controls were recruited from SGH General Ophthalmology clinics or the general population. Variations in sample size, reported throughout the manuscript, were due to some patients having performed some of the assessments at other hospitals or missing data (Supplementary Fig. 1).

Patients were eligible if they had a PA causing radiological optic chiasm indentation or distortion and were scheduled for surgical resection by a neurosurgeon. Patients and controls were aged 21 years or older. Exclusion included a history of retinopathy and/or optic neuropathy, raised intraocular pressure or glaucoma in either eye; refractive error exceeding ± 6.0 DSph or ± 3.0 DCyl; previous intraocular surgery (except uncomplicated cataract procedures); cataract severity worse than NS2+; participants on any drugs that may affect pupillary size or responses (e.g. Pilocarpine, Atropine), or having conditions affecting afferent or efferent pupillary pathways (e.g. Horner’s syndrome, Adie’s tonic pupil); and participants with previous trauma to the eyes or previous intraocular inflammation.

### Study assessments

All patients underwent pre-operative (7.0 ± 5.4 weeks before surgery) and post-operative (12.0 ± 1.5 weeks after surgery) assessments, including clinical ophthalmic evaluations, MRI and pupillometry. Healthy controls underwent a single baseline assessment, consisting of ophthalmic evaluations and pupillometry.

### Clinical evaluation and imaging

#### Demographics and medical history

The data collected included age, gender, ethnicity, medical history (e.g. diabetes, systemic conditions), ocular history, medication use and smoking status.

#### Ophthalmic assessments

All participants underwent a comprehensive ophthalmic evaluation including measurement of presenting visual acuity (VA) defined as LogMAR visual acuity measured using the patient’s best correction with their habitual optical correction. Additional assessments included RNFL imaging using high-definition OCT and visual field testing using standard automated perimetry [Humphrey Visual Field (HVF) analyser].

#### MRI assessment

Pre-operative and post-operative MRI scans were obtained for all patients except two who lacked pre-operative scans due to assessments at another hospital. Fine-cut coronal T_2_-weighted MRI scans were used to assess the optic chiasm upward displacement and thickness. All MRI evaluations were conducted by a neurosurgeon, who extracted and measured optic chiasm displacement and thickness measurements to the nearest 0.1 mm. The optic chiasm was identified by tracing bilateral optic nerves backwards from the retina, through the intraconal portion into the optic canal, and then subsequently superior to the pituitary tumour. Three measurements were taken for each parameter on each MRI, and their average was used for analysis.

### Handheld chromatic pupillometry

**Figure 1:**
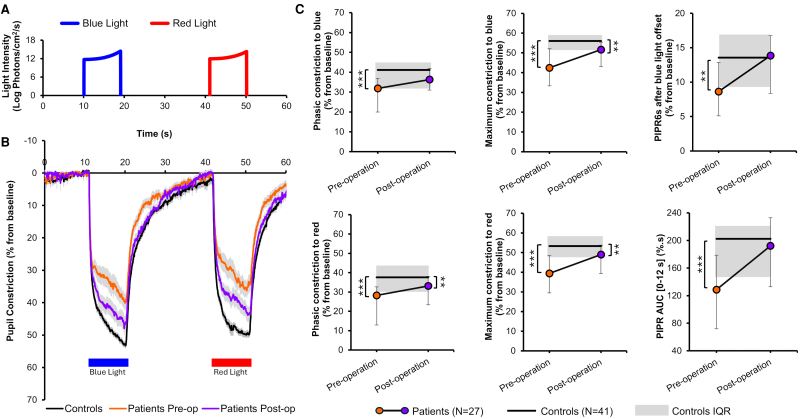
**Average baseline-adjusted pupillary light responses in patients with pituitary adenoma (*n* *=* 27) and controls (*n* *=* 41).** (**A**) Pupillometry light protocol. (**B**) Mean pupillary responses to blue and red light in patients with pituitary adenoma (*n* *=* 27) before and after surgery, and controls. Data are plotted as average ± SE. (**C**) Differences in the median of the main pupillometric features in patients with pituitary adenoma and controls. Statistical comparisons between groups were performed using a Mann–Whitney *U* test. Error bars represent IQR and connecting lines illustrate individual changes. **P *<* 0.01; ***P *<* 0.001. AUC, area under the curve; IQR, interquartile range; PIPR, post-illumination pupillary response; Post-op, post-operation; Pre-op, pre-operation.

Pupillometry testing was conducted using a standardized 1-min protocol with a custom-built handheld chromatic pupillometer in a darkened room (<1 lux),^33^ aligning with recommended practices.^34^ The device, designed for monocular use, included a silicone rubber eye cup for comfortable positioning over the study eye and ensured light isolation, with the fellow eye covered by the participant’s hand.^17^ The protocol involved five consecutive phases: 10 s of darkness for baseline pupil size measurement, 9 s of exponentially increasing blue light stimulation (11.7–14.4 Log photons/cm^2^/s; λmax = 469 nm, FWHM = 33 nm), 22 s of darkness for pupillary redilation, 9 s of exponentially increasing red light stimulation (11.9–14.3 Log photons/cm^2^/s; λmax = 640 nm, FWHM = 17 nm) and a final 10 s of darkness to assess redilation^17-19,35^ (Fig. 1A). Participants fixated on a central dim red zone (<0.1 lux) within the device, and any issues with fixation or excessive blinking prompted a repeat of the test. An infrared camera positioned at ∼60° below the lower eyelid recorded horizontal pupil size changes at a frame rate of 30 fps.

The same eye was consistently tested pre- and post-operatively for each patient. When complete pupillometric and ophthalmic data were available for both eyes, the tested eye was randomly selected. In cases where data were missing for one eye, the eye with complete data was included in the analysis.

### Data analysis and statistics

Pupil radius measurements were processed using a semi-automated algorithm for blink artefact removal and expressed as a percentage change from baseline pupil size. Sixteen pupillometric features were extracted from individual blink-free traces for further analysis, among which we have phasic constriction to blue (Phasic-Blue) and red (Phasic-Red) light, maximum constriction to blue (Max-Blue) and red (Max-Red) light, post illumination pupillary response 6 (PIPR6s) and 12 s after light offset (PIPR12s), PIPR area under the curve (PIPR AUC 0–12 s) and redilation slope 1.7 s following light offset (PIPR > 1.7 slope).

All pupillometric, MRI and ophthalmic features are reported as median [interquartile range (IQR)] and were compared between controls and PA patients using the Mann–Whitney *U* test. Comparisons of pre- and post-operative outcomes in PA patients were performed using the Wilcoxon signed-rank test. Associations between structural and functional outcomes were assessed using Spearman’s rank correlation coefficient. Demographic data are presented as median (IQR) or number (%) and were compared between controls and PA groups using either the Mann–Whitney *U* test or *χ*^2^ test, as appropriate. Recovery was defined as the point at which a patient’s pupillometric or ophthalmic features returned within the 95% confidence interval (CI) of control values. All statistical analyses were conducted using SPSS Statistics v26 (IBM, USA).

## Results

### Demographics and clinical characteristics of participants

Data from 41 healthy controls [median age = 53.6 (IQR = 13.7) years, 46.3% males, 97.6% ethnic Chinese] and 27 PA patients [55.4 years (23.4), 40.7% males, 74.1% ethnic Chinese] were analysed. Groups were age-matched (*P* *=* 0.72). The control group had a higher proportion of Chinese participants (*P* *=* 0.03). Other baseline clinical characteristics, including diabetes, cataract history and prior posterior chamber intraocular lens (PCIOL) implantation, were similar between groups (all *P* *>* 0.05). A detailed breakdown of demographic and clinical characteristics for the total sample is presented in Table 1, while subgroup characteristics are provided in Supplementary Tables 1–3.

**Table 1:** Demographics and clinical characteristics of patients with pituitary adenoma (*n* *=* 27) and healthy controls (*n* *=* 41)

Demographic and clinical characteristics	Controls	Patients with pituitary adenoma	*P*-value
*N*	41	27	
Age, median (IQR), years	53.6 (13.7)	55.4 (23.4)	0.72
Gender, male, no. (%)	19 (46.3)	11 (40.7)	0.65
Ethnicity			0.03
Chinese, no. (%)	40 (97.6)	20 (74.1)	
Indian, no. (%)	1 (2.4)	3 (11.1)	
Malay, no. (%)	0	1 (3.7)	
Others, no. (%)	0	3 (11.1)	
Diabetes, no. (% with)	1 (2.4)	1 (3.7)	0.76
Cataract, no. (% with)	18 (43.9)	18 (66.7)	0.07
PCIOL, no. (% with)	2 (4.9)	2 (7.4)	0.67

Mann–Whitney *U* test was used to compare age between controls and pituitary adenoma groups. *χ*^2^ test was used to compare all other variables between the two groups.

IQR, interquartile range; PCIOL, posterior chamber intraocular lens.

### Pre-operative pupillary light responses in patients with pituitary adenoma

Prior to TSS, patients with PA exhibited significant differences in the baseline-adjusted pupillary responses to both blue and red light stimuli compared to healthy controls (Fig. 1B). Patients with PA demonstrated prolonged constriction latency [controls: 0.35 s (IQR: 0.17), patients: 0.48 s (0.31) for blue light; controls: 0.47 s (0.21), patients: 0.58 s (0.82) for red light], reduced phasic constriction [controls: 41.1% (12.6), patients: 31.9% (16.9) for blue; controls: 37.6% (12.7), patients: 28.3% (19.7) for red light] and lower maximum constriction [controls: 56.0% (6.8), patients: 42.4% (18.8) for blue light; controls: 53.3% (9.8), patients: 39.4% (18.9) for red light] (all *P* *<* 0.001 for both stimuli). Pupillary redilation following blue light exposure was also impaired, with diminished PIPR at 6 s [controls: 13.6% (7.5), patients: 8.6% (7.8), *P* *=* 0.001] and 12 s [controls: 6.4% (8.3), patients: 2.8% (5.3), *P* *=* 0.04] after blue light offset, a significantly reduced PIPR AUC 0–12 s [controls: 202.5%.s (79.4), patients: 128.6%.s (106.8), *P* *<* 0.001] compared to controls (Fig. 1C) and a smaller PIPR > 1.7 slope [controls: −1.0%/s (0.6), patients: −0.7%/s (0.5), *P* *=* 0.001] (Supplementary Fig. 2 and Table 4).

### Recovery in pupillometric features following surgical decompression

Following TSS, constriction latency improved but remained higher than controls [controls: 0.35 s (0.17), patients: 0.40 s (0.26) for blue light; controls: 0.47 s (0.21), patients: 0.56 s (0.30) for red], though this difference was not statistically significant. Maximum constriction to both stimuli increased post-operatively but did not return to control levels [controls: 56.0% (6.8), patients: 51.6% (12.4), *P* *=* 0.007 for blue; controls: 53.3% (9.8), patients: 49.0% (14.4), *P* *=* 0.01 for red]. In contrast, blue PIPR6s [controls: 13.6% (7.5), patients: 13.9% (8.4)] and PIPR AUC 0–12 s [controls: 202.5%.s (79.4), patients: 192.3%.s (100.1)] normalized post-surgery, falling within the 95% CI of control (Fig. 1C; Supplementary Table 4).

### Associations between structural and functional recovery following surgery

**Figure 2:**
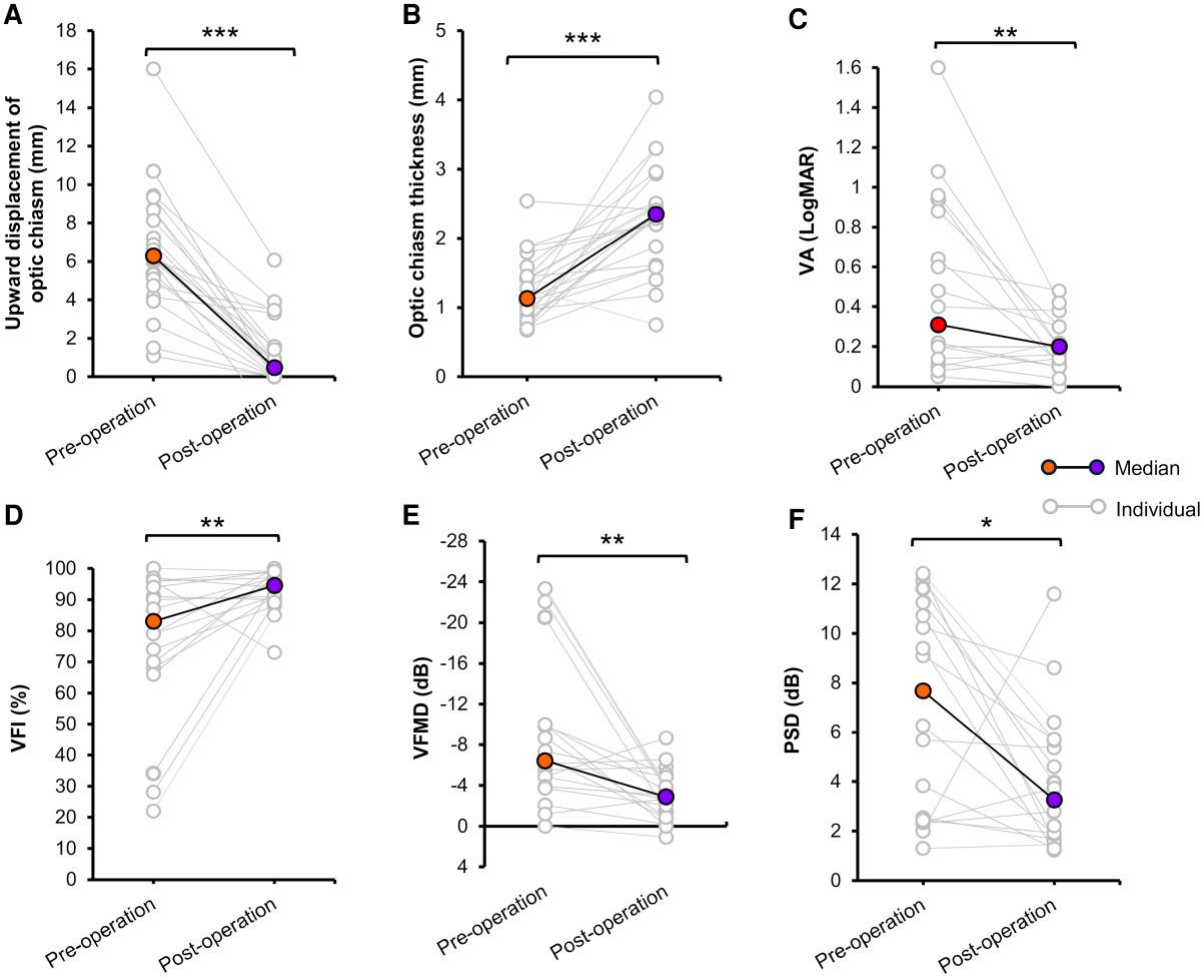
**Paired comparison of pre-operative and post-operative patient outcomes.** (**A, B**) Structural MRI outcomes (*n* *=* 25). (**C**) Visual acuity (VA) (*n* *=* 18). (**D–F**) Humphrey Visual Field (HVF) outcomes (*n* *=* 18). Statistical comparisons pre- and post-surgery were performed using Wilcoxon signed-rank test. *P *<* 0.05; ** *P* *<* 0.01; ***P *<* 0.001. dB, decibels; LogMAR, logarithm of minimum angle of resolution; mm, millimetre; PSD, pattern SD; VA, visual acuity; VFI, visual field index; VFMD, visual field mean deviation.

**Figure 3:**
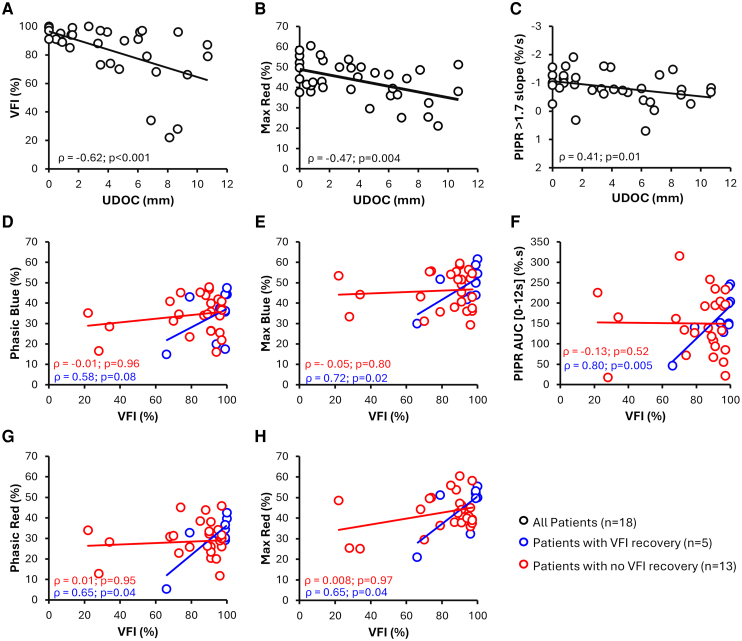
**Scatter plots showing Spearman correlations.** Each point represents a patient’s pre- or post-operative measurement. (**A–C**) Correlations between structural (UDOC) and functional (VFI, Max-Red, PIPR > 1.7 slope) outcomes. (**D–H**) Correlations between functional ocular (VFI) and functional pupillometric outcomes. AUC, area under the curve; Max-Red, maximum constriction to red light; PIPR > 1.7 slope, post-illumination pupillary response at >1.7 slope; UDOC, upward displacement of the optic chiasm; VFI, visual field index.

Paired comparisons of MRI features before and after surgery showed significant reductions in the upward displacement of the optic chiasm (UDOC) [pre-op: 6.3 mm (3.8), post-op: 0.8 mm (1.6), *P* *<* 0.001; Fig. 2A) and an increase in optic chiasm thickness (OpCT) [pre-op: 1.1 mm (0.5), post-op: 2.4 mm (0.6), *P* < 0.001; Fig. 2B], indicating effective structural decompression. OCT data from 17 patients showed no difference in RNFL thickness pre- [88 (9) µm] versus post-surgery [86 (5) µm; *P* *>* 0.05]. However, RNFL thickness remained significantly thinner compared with controls [96 (5) µm; *P* *<* 0.05 for both pre- and post-surgery comparisons]. Comparatively, functional recovery was also noticed post-surgery. VA, as well as visual field outcomes such as the visual field index (VFI), visual field mean deviation (VFMD) and pattern standard deviation (PSD), improved post-surgery (all *P* *<* 0.05; Fig. 2C–F) but did not reach control levels (Supplementary Table 1 and Fig. 3).

Spearman correlation analysis revealed a significant negative correlation between UDOC and VFI (*ρ* = −0.62, *P* *<* 0.001; Fig. 3A) as well as UDOC and Max-Red (*ρ* = −0.47, *P* *=* 0.004; Fig. 3B). Conversely, UDOC was positively correlated with PIPR > 1.7 slope (*ρ* = 0.41, *P* *=* 0.01; Fig. 3C). In contrast, changes in UDOC were not correlated with changes in VA (*ρ* = 0.30, *P* *=* 0.08). Similarly, Max-Red and VA were not correlated (*ρ* = −0.11, *P* *=* 0.51), while Max-Red and VFI showed a marginal correlation that did not reach statistical significance (*ρ* = 0.32, *P* *=* 0.06). A comprehensive overview of all correlations is provided in the Supplementary material (Supplementary Fig. 4).

Subgroup analyses classified patients based on whether their VFI fell within the 95% CI of healthy controls, with those meeting this threshold considered to have achieved full recovery. Although no pupillometric feature was significantly correlated with VFI in all 18 participants, stratified analysis revealed that several pupillometric features, including Phasic-Red (Fig. 3G), Max-Blue and Max-Red and PIPR AUC 0–12 s, were significantly correlated with VFI in patients who achieved full recovery (*n* *=* 5; all *P* *<* 0.05), but not in those who did not (*n* *=* 13; all *P* *>* 0.05) (Fig. 3E–H).

### Visual field recovery and pupillometric features

**Figure 4:**
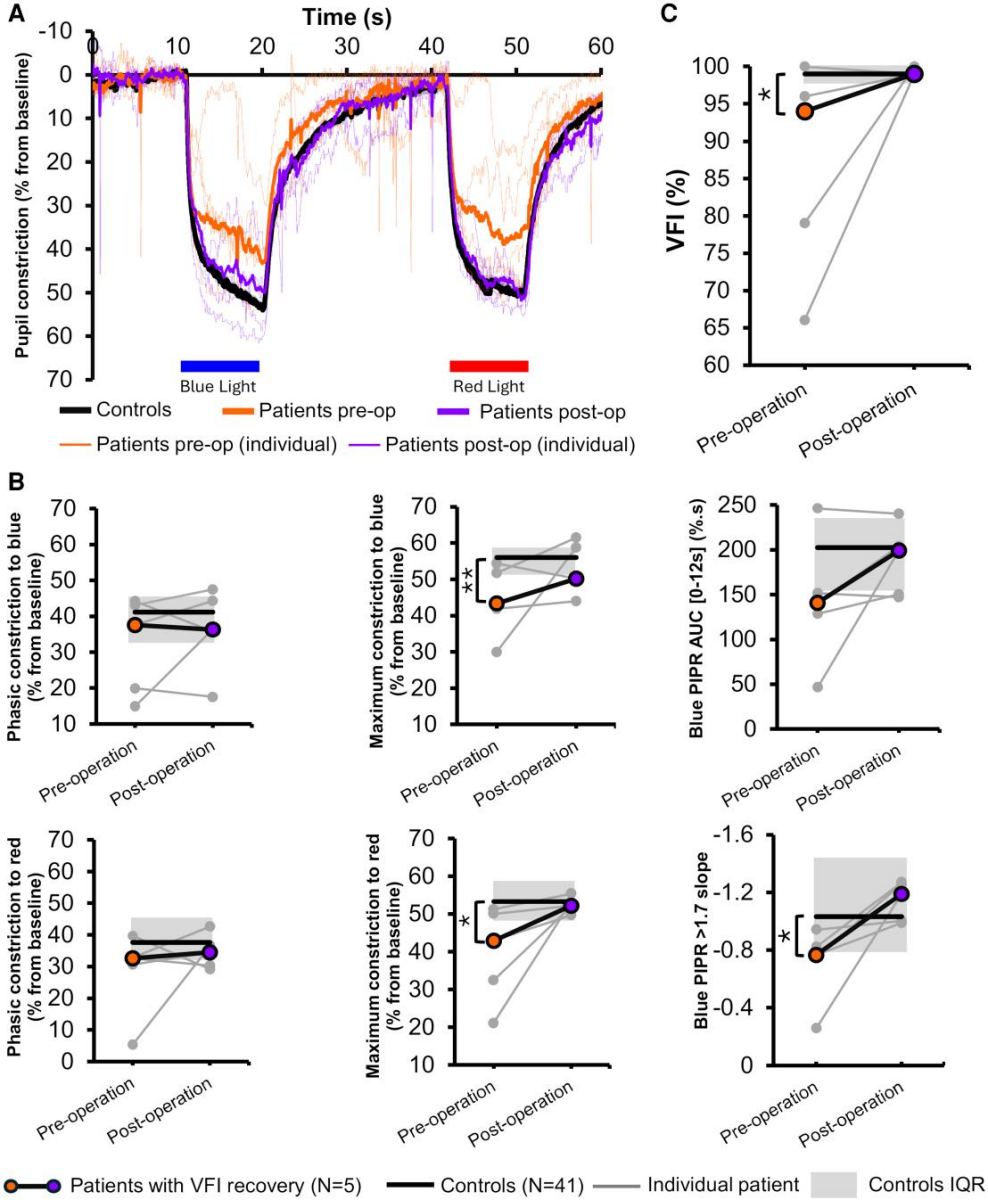
**PLR and comparisons of pupillometric parameters in patients with VFI recovery.** (**A**) Mean pupil response traces to blue and red light stimuli, in patients with pituitary adenoma with VFI recovery (*n* *=* 5) before (orange) and after (purple) surgery and controls (black). Lighter orange and purple represent the individual plots of patients before and after surgery, respectively. (**B**) Differences in main pupillometric features between patients pre- and post-operation and controls. (**C**) Difference in VFI in patients with PA before and after surgery. Individual data points are represented in grey. For **B** and **C**, since median values are presented, the individual data points of one of the participants may be masked by the median trace. Statistical comparisons between groups were performed using a Mann–Whitney *U* test. *P *<* 0.05; ** *P* *<* 0.01. AUC, area under the curve; IQR, interquartile range; VFI, visual field index; PIPR, post-illumination pupillary response; PLR, pupillary light response; Post-op, post-operation; Pre-op, pre-operation.

Patients with and without visual field recovery post-surgery exhibited distinct trajectories in PLR parameters. Patients who achieved visual field recovery demonstrated post-surgical phasic constriction and maximum constriction at control levels (*P* *>* 0.05 for both lights; Fig. 4). In contrast, patients without visual field recovery exhibited persistent deficits, including a 5.9% reduction in Phasic-Red (*P* *=* 0.03) and reduction in Max-Blue (4.4%) and Max-Red (10.8%) (both *P* *=* 0.01) compared to controls (Supplementary Fig. 5).

Despite these group differences in phasic and maximum constriction, post-surgery pupillometric redilation features (i.e. blue PIPR6s, blue PIPR12s, PIPR AUC 0–12 s and PIPR > 1.7 slope) fell within the 95% CI of control values in both recovered and non-recovered patients (all *P* *>* 0.05).

Individual cases showing complete (Supplementary Fig. 6), partial (Supplementary Fig. 7) and limited (Supplementary Fig. 8) structural and functional recovery are reported in Supplementary material.

### Enhanced detection of pupillary deficits using chromatic pupillometry

Pre-operatively, clinical examination revealed no relative afferent pupillary defect (RAPD) in 11 of 18 patients; however, all of these patients exhibited pupillometric abnormalities when assessed with direct monocular HCP. Following surgery, 16 patients demonstrated no clinical RAPD. Of these, nine continued to show residual pupillometric defects on HCP. Among the 16 patients without clinical RAPD post-operatively, 5 achieved complete visual field recovery, and 4 of these patients also demonstrated full recovery on HCP.

## Discussion

This study evaluated visual and non-visual pupillometric functions in PA before and after TSS. We demonstrated HCP to be a feasible, objective, and non-invasive tool for assessing the functional disruption and restoration of the visual and non-visual pupillary pathways. Notably, irrespective of the visual outcomes, PA surgery resulted in consistent improvements in PLR, aligning with structural and functional recovery. Importantly, melanopsin-driven responses normalized after surgery even in individuals without visual field recovery, indicating that melanopsin-mediated pupillary pathways may restore independently of vision.

In line with previous studies,^12,13^ PA patients exhibited significant pre-operative impairments in pupillary responses to blue and red light stimuli, suggesting dysfunction in both intrinsic (melanopsin-driven) and extrinsic (cone-mediated) pathways due to optic chiasm compression. Pupillometric dysfunction in PA aligns with previous findings in glaucoma^17,36^ and diabetic retinopathy,^19^ diseases affecting ipRGC integrity. The optic chiasm compression in PA can lead to retrograde degeneration of retinal ganglion cells,^37^ presented on OCT as thinning of the RNFL and ganglion cell layer in affected patients.^38^

Post-operative MRI confirmed effective decompression of the optic chiasm, with reduced chiasmal displacement and increased chiasm thickness. However, functional outcomes varied considerably among patients, showing that structural restoration does not always equate to complete functional recovery. This variability is consistent with prior studies demonstrating that post-surgical visual recovery is influenced by factors such as the degree of pre-operative damage, duration of symptoms and individual neuroplasticity.^39-41^ Although some patients exhibited near-normalization of PLR, others showed persistent deficits despite apparent structural improvements on MRI. These findings emphasize the need for a functional assessment of visual pathways in addition to structural imaging, as anatomical normalization alone may not fully capture the extent of neuro-ophthalmic recovery. Additionally, VFI correlated well with structural features and showed a trend for correlation with pupillometry, supporting its use as a more relevant functional outcome in PA compared to visual acuity. Notably, the full field chromatic pupillometry protocol used here may not fully capture subtle localized retinal or visual field-specific dysfunction, particularly in cases of sectoral or hemifield loss. Spatially resolved approaches, such as multifocal pupillography^42^ or chromatic pupil campimetry,^43^ could therefore provide complementary insights by capturing regional pupillary responses aligned with topographic visual field defects.

A key finding of this study is the differential recovery observed between melanopsin-driven and cone-mediated responses. While PIPR to blue light (i.e. PIPR6s, PIPR AUC 0–12 s, PIPR > 1.7 slope), primarily mediated by melanopsin,^44^ and reduced in patients with glaucoma,^17^ fully recovered, maximum and phasic constriction, predominantly cone-mediated, remained impaired. These findings suggest that intrinsic ipRGC pathways may be more resilient to compressive damage and capable of greater functional recovery than extrinsic cone-mediated pathways.^45^ This differential recovery may reflect both anatomical and molecular properties of ipRGC subtypes. M1 ipRGCs, rich in melanopsin, show strong intrinsic photosensitivity and are known to resist optic nerve injury more effectively than other subtypes.^46^ In contrast, M2 ipRGCs receive substantial extrinsic input from cones and bipolar cells^47^ and appear more vulnerable to structural damage.^48^ Both M1 and M2 project to the olivary pretectal nucleus and contribute to the PLR,^49^ but their differential resilience may account for the partial restoration observed in our study. While recovery of PIPR likely reflects the robustness of M1 ipRGCs, the persistence of deficits in maximum and phasic constriction may result from residual dysfunction in cone pathways, disrupted synaptic integration or damage to more vulnerable ipRGC subtypes such as M2. Whether the selective ipRGC recovery observed represents true neuroregeneration or compensatory mechanisms remains unclear and warrants further investigation using approaches that can differentiate ipRGC subtypes and their specific projections. Conversely, direct cone dysfunction in PA is not well established in the literature; it is possible that cone pathways are secondarily affected by RGC degeneration. Experimental models have shown that long-term ischaemic damage can extend beyond inner retinal layers to affect outer retinal structures, including cone pathways.^50^ Moreover, ipRGC degeneration could lead to neurovascular alterations that indirectly impact cone function, and chronic compression has been shown to trigger retinal remodelling, involving neuronal cell death and synaptic rewiring.^51^

Correlations between visual field recovery and PLR improvement further reinforce the potential role of pupillometry as a biomarker for neuro-ophthalmic recovery following TSS. Patients with greater VFI recovery showed enhanced PLR, particularly in phasic and maximum constriction. Given that perimetry relies on subjective patient responses and requires sustained attention,^8^ pupillometry offers an objective and quantifiable alternative for assessing visual function. Furthermore, direct HCP seems to provide a sensitive approach for detecting and monitoring functional deficits in patients with PA, providing a complementary, objective support to conventional RAPD assessment. This study is the largest to date investigating pupillometry in PAs, yet some limitations should be acknowledged. First, the sample size remains relatively small for predictive modelling and larger cohorts are needed to strengthen statistical power and validate pupillometric predictors of visual recovery. Second, some MRI, visual field and OCT data were missing due to logistical issues, reducing the number of complete cases and leading to stratification. Additionally, unilateral analysis may have overlooked inter-eye asymmetries, particularly in patients with asymmetric optic nerve or chiasmal compression. Third, pupillary responses measured in our study may not strictly reflect the laboratory-defined PIPR, which typically requires pharmacological dilation, brief and intense light stimuli and shorter exposure durations.^44,52^ Nevertheless, similar pupillometric features, obtained using the same HCP device, have previously been shown to be reduced in glaucoma patients, a population known to exhibit impaired ipRGC function.^17^ Additionally, cataract and cataract surgery can influence circadian and sleep-related outcomes, particularly depending on intraocular lens type^53^; however, mild cataracts appear to have minimal impact on non-visual pupillometric responses under comparable light stimulation.^54^ Nevertheless, the inclusion of participants with mild cataract and prior cataract surgery may introduce residual inter-individual variability in ocular light transmission, which should be addressed in larger, dedicated studies. Finally, our protocol was limited to a short follow-up period of ∼12 weeks, which may have missed long-term functional changes following surgery.

In conclusion, our findings demonstrate that although TSS for PAs leads to significant structural decompression of the optic chiasm, functional recovery of the PLR remains incomplete in certain cases. Notably, ipRGC-mediated responses, measured by the PIPR to blue light, fully recover post-operatively, whereas cone-mediated pathways, assessed by maximum and phasic constriction, show persistent impairments. The preservation and restoration of melanopsin-driven ipRGC responses are especially notable, as these pathways play a critical role in circadian entrainment, sleep regulation and other systemic processes.^55^ HCP thus provides a sensitive, objective and non-invasive biomarker of recovery, correlating with visual field outcomes and complementing conventional neuro-ophthalmic assessments. Future studies integrating pupillometry with validated sleep measures, circadian phenotyping and patient-reported outcomes will be essential to determine the clinical relevance of melanopsin recovery and to establish whether pupillometry can serve as a biomarker of broader systemic outcomes in pituitary disease.

## Supplementary Material

fcag233_Supplementary_Data

## Data Availability

The de-identified datasets and the study protocol can be made available from the corresponding author upon reasonable request.

## References

[1] Wang MTM, Meyer JA, Danesh-Meyer HV. Neuro-ophthalmic evaluation and management of pituitary disease. Eye (Lond). 2024;38(12):2279–2288. 39039214 10.1038/s41433-024-03187-x PMC11306754

[2] Mortini P, Losa M, Barzaghi R, Boari N, Giovanelli M. Results of transsphenoidal surgery in a large series of patients with pituitary adenoma. Neurosurgery. 2005;56(6):1222–1233; discussion 1233. 15918938 10.1227/01.neu.0000159647.64275.9d

[3] Layard Horsfall H, Lawrence A, Venkatesh A, et al Reported outcomes in transsphenoidal surgery for pituitary adenomas: A systematic review. Pituitary. 2023;26(2):171–181. 36862265 10.1007/s11102-023-01303-w PMC10247847

[4] Cohen AR, Cooper PR, Kupersmith MJ, Flamm ES, Ransohoff J. Visual recovery after transsphenoidal removal of pituitary adenomas. Neurosurgery. 1985;17(3):446–452. 4047355 10.1227/00006123-198509000-00008

[5] Rowe FJ, Cheyne CP, García-Fiñana M, et al Detection of visual field loss in pituitary disease: Peripheral kinetic versus central static. Neuroophthalmology. 2015;39(3):116–124. 27928344 10.3109/01658107.2014.990985 PMC5123138

[6] Lee IH, Miller NR, Zan E, et al Visual defects in patients with pituitary adenomas: The myth of bitemporal hemianopsia. AJR Am J Roentgenol. 2015;205(5):W512–W518. 26496573 10.2214/AJR.15.14527

[7] Danesh-Meyer HV, Wong A, Papchenko T, et al Optical coherence tomography predicts visual outcome for pituitary tumors. J Clin Neurosci. 2015;22(7):1098–1104. 25891894 10.1016/j.jocn.2015.02.001

[8] Rai BB, Sabeti F, Carle CF, Maddess T. Visual field tests: A narrative review of different perimetric methods. J Clin Med. 2024;13(9):9. 10.3390/jcm13092458 PMC11084906 38730989

[9] Carter J. Visual evoked potentials, Clinical Neurophysiology. Oxford Academic; 2016:567–578.

[10] Hsu CH, Kuo LT. Application of pupillometry in neurocritical patients. J Pers Med. 2023;13(7):1100. 37511713 10.3390/jpm13071100 PMC10381796

[11] Kim TJ. Quantitative assessments of pupillary light reflexes in neurocritically ill patients. Neurocrit Care. 2022;15(2):79–87.

[12] Lenga P, Jakobs M, Jesser J, Trong PD, Unterberg AW, Beynon C. The use of quantitative pupillometry in patients with pituitary tumors: A technical note. Acta Neurochir (Wien). 2022;164(6):1599–1604. 35445853 10.1007/s00701-022-05214-w PMC9160135

[13] Lenga P, Grutza M, Kühlwein D, Walter J, Krieg SM, Beynon C. Evaluating optic system compression in sellar tumors: A novel application of quantitative pupillometry. Acta Neurochir (Wien). 2024;166(1):510. 39731604 10.1007/s00701-024-06401-7 PMC11682009

[14] Gooley JJ, Mien IH, Hilaire MAS, et al Melanopsin and rod–cone photoreceptors play different roles in mediating pupillary light responses during exposure to continuous light in humans. J Neurosci. 2012;32(41):14242–14253. 23055493 10.1523/JNEUROSCI.1321-12.2012 PMC3515688

[15] Rukmini AV, Milea D, Gooley JJ. Chromatic pupillometry methods for assessing photoreceptor health in retinal and optic nerve diseases. Front Neurol. 2019;10:76. 30809186 10.3389/fneur.2019.00076 PMC6379484

[16] Chougule PS, Najjar RP, Finkelstein MT, Kandiah N, Milea D. Light-induced pupillary responses in Alzheimer’s disease. Front Neurol. 2019;10:360. 31031692 10.3389/fneur.2019.00360 PMC6473037

[17] Najjar R, Dhara R, Finkelstein M, et al Handheld chromatic pupillometry can accurately and rapidly reveal functional loss in glaucoma. Br J Ophthalmol. 2023;107:663–670. 34853018 10.1136/bjophthalmol-2021-319938 PMC10176376

[18] Finkelstein MT, Nongpiur ME, Husain R, et al Handheld chromatic pupillometry can reliably detect functional glaucomatous damage in eyes with high myopia. Br J Ophthalmol. 2023;108:818–825. 10.1136/bjo-2023-323878 37524446

[19] Tan T, Finkelstein MT, Tan GSW, et al Retinal neural dysfunction in diabetes revealed with handheld chromatic pupillometry. Clin Exp Ophthalmol. 2022;50(7):745–756. 35616273 10.1111/ceo.14116 PMC9796882

[20] Edelmayer MV, Strasser T, Jung R, et al Chromatic pupil campimetry as objective diagnostic tool for progressive optic neuropathies. Doc Ophthalmol. 2025. 10.1007/s10633-025-10054-x 41094347

[21] Kelbsch C, Stingl K, Jung R, et al How lesions at different locations along the visual pathway influence pupillary reactions to chromatic stimuli. Graefes Arch Clin Exp Ophthalmol. 2022;260(5):1675–1685. 34902059 10.1007/s00417-021-05513-5 PMC9007757

[22] Abbott SM. The eyes have it: Pupillary assessment as a measure of sleep and circadian health. Sleep. 2025;48(2):zsae285. 39699128 10.1093/sleep/zsae285 PMC11807885

[23] Fernandez DC, Chang YT, Hattar S, Chen SK. Architecture of retinal projections to the central circadian pacemaker. Proc Natl Acad Sci U S A. 2016;113(21):6047–6052. 27162356 10.1073/pnas.1523629113 PMC4889372

[24] Meng J, Huang X, Ren C, Xue T. Non-image-forming functions of intrinsically photosensitive retinal ganglion cells. Annu Rev Neurosci. 2025;48:211–229. 40053827 10.1146/annurev-neuro-112723-035532

[25] Příhodová I, Nepožitek J, Kelifová S, et al Subjective and polysomnographic evaluation of sleep in mitochondrial optic neuropathies. J Sleep Res. 2021;30(2):e13051. 32524698 10.1111/jsr.13051

[26] Tegegne YB, Hussen MS, Ayele FA, Mersha GA. Association of glaucoma with poor quality of sleep in an Ethiopian glaucoma population—A comparative cross-sectional study. Clin Ophthalmol. 2022;16:3701–3710. 36389639 10.2147/OPTH.S387623 PMC9661991

[27] Gracitelli CPB, Duque-Chica GL, Roizenblatt M, et al Intrinsically photosensitive retinal ganglion cell activity is associated with decreased sleep quality in patients with glaucoma. Ophthalmology. 2015;122(6):1139–1148. 25858174 10.1016/j.ophtha.2015.02.030

[28] Wang H, Zhang Y, Ding J, Wang N. Changes in the circadian rhythm in patients with primary glaucoma. PLoS One. 2013;8(4):e62841. 23658653 10.1371/journal.pone.0062841 PMC3639222

[29] Qiu M, Ramulu PY, Boland MV. Association between sleep parameters and glaucoma in the United States population: National health and nutrition examination survey. J Glaucoma. 2019;28(2):97–104. 30585942 10.1097/IJG.0000000000001169

[30] Lin MR, Chen PY, Wang HC, Lin PC, Lee HC, Chiu HY. Prevalence of sleep disturbances and their effects on quality of life in adults with untreated pituitary tumor and meningioma. J Neurooncol. 2021;154(2):179–186. 34304334 10.1007/s11060-021-03811-w

[31] Jimura H, Yoshikawa T, Obayashi K, Miyata K, Saeki K, Ogata N. Post-illumination pupil response and sleep quality in patients with glaucoma: The LIGHT study. Invest Ophthalmol Vis Sci. 2023;64(12):34. 10.1167/iovs.64.12.34 PMC10516763 37728904

[32] Rach H, Kilic-Huck U, Reynaud E, et al The melanopsin-mediated pupil response is reduced in idiopathic hypersomnia with long sleep time. Sci Rep. 2022;12(1):9018. 35637236 10.1038/s41598-022-13041-3 PMC9151765

[33] Milea D, Najjar R, Aung T. Hand held ophthalmic and neurological screening device. Published online December 23, 2021:US20210393122A1. Accessed August 30, 2025. https://patents.google.com/patent/US20210393122A1/en

[34] Kelbsch C, Strasser T, Chen Y, et al Standards in pupillography. Front Neurol. 2019;10:129. 30853933 10.3389/fneur.2019.00129 PMC6395400

[35] Finkelstein MT, Najjar RP, Chougule P, Mathur R, Milea D. Chromatic pupillometry in multiple evanescent white dot syndrome masquerading as atypical optic neuritis. Acta Ophthalmol. 2022;100(6):713–715. 34411454 10.1111/aos.14998

[36] Kankipati L, Girkin CA, Gamlin PD. The post-illumination pupil response is reduced in glaucoma patients. Invest Ophthalmol Vis Sci. 2011;52(5):2287–2292. 21212172 10.1167/iovs.10-6023 PMC3080733

[37] Keller J, Sánchez-Dalmau BF, Villoslada P. Lesions in the posterior visual pathway promote trans-synaptic degeneration of retinal ganglion cells. PLoS One. 2014;9(5):e97444. 24857938 10.1371/journal.pone.0097444 PMC4032251

[38] Shinohara Y, Todokoro D, Yamaguchi R, Tosaka M, Yoshimoto Y, Akiyama H. Retinal ganglion cell analysis in patients with sellar and suprasellar tumors with sagittal bending of the optic nerve. Sci Rep. 2022;12(1):11092. 35773336 10.1038/s41598-022-15381-6 PMC9246971

[39] Dutta P, Gyurmey T, Bansal R, et al Visual outcome in 2000 eyes following microscopic transsphenoidal surgery for pituitary adenomas: Protracted blindness should not be a deterrent. Neurol India. 2016;64(6):1247–1253. 27841194 10.4103/0028-3886.193829

[40] Ho RW, Huang HM, Ho JT. The influence of pituitary adenoma size on vision and visual outcomes after trans-sphenoidal adenectomy: A report of 78 cases. J Korean Neurosurg Soc. 2015;57(1):23–31. 25674340 10.3340/jkns.2015.57.1.23 PMC4323501

[41] Sabel BA, Gao Y, Antal A. Reversibility of visual field defects through induction of brain plasticity: Vision restoration, recovery and rehabilitation using alternating current stimulation. Neural Regen Res. 2020;15(10):1799–1806. 32246620 10.4103/1673-5374.280302 PMC7513964

[42] Maddess T, Bedford SM, Goh XL, James AC. Multifocal pupillographic visual field testing in glaucoma. Clin Exp Ophthalmol. 2009;37(7):678–686. 19788664 10.1111/j.1442-9071.2009.02107.x

[43] Kelbsch C, Lange J, Wilhelm H, et al Chromatic pupil campimetry reveals functional defects in exudative age-related macular degeneration with differences related to disease activity. Transl Vis Sci Technol. 2020;9(6):5. 10.1167/tvst.9.6.5 PMC7409006 32821502

[44] Adhikari P, Feigl B, Zele AJ. Rhodopsin and melanopsin contributions to the early redilation phase of the post-illumination pupil response (PIPR). PLoS One. 2016;11(8):e0161175. 27548480 10.1371/journal.pone.0161175 PMC4993463

[45] Ventura LM, Venzara FX, Porciatti V. Reversible dysfunction of retinal ganglion cells in non-secreting pituitary tumors. Doc Ophthalmol. 2009;118(2):155–162. 18670795 10.1007/s10633-008-9143-8 PMC3707133

[46] Pérez de Sevilla Müller L, Sargoy A, Rodriguez AR, Brecha NC. Melanopsin ganglion cells are the most resistant retinal ganglion cell type to axonal injury in the rat retina. PLoS One. 2014;9(3):e93274. 24671191 10.1371/journal.pone.0093274 PMC3966869

[47] Patterson SS, Neitz M, Neitz J. S-cone circuits in the primate retina for non-image-forming vision. Semin Cell Dev Biol. 2022;126:66–70. 33994300 10.1016/j.semcdb.2021.05.004 PMC8589871

[48] Duan X, Qiao M, Bei F, Kim IJ, He Z, Sanes JR. Subtype-specific regeneration of retinal ganglion cells following axotomy: Effects of osteopontin and mtor signaling. Neuron. 2015;85(6):1244–1256. 25754821 10.1016/j.neuron.2015.02.017 PMC4391013

[49] Tapia ML, Nascimento-dos-Santos G, Park KK. Subtype-specific survival and regeneration of retinal ganglion cells in response to injury. Front Cell Dev Biol. 2022;10:956279. 36035999 10.3389/fcell.2022.956279 PMC9411869

[50] Palmhof M, Frank V, Rappard P, et al From ganglion cell to photoreceptor layer: Timeline of deterioration in a rat ischemia/reperfusion model. Front Cell Neurosci. 2019;13:174. 31133806 10.3389/fncel.2019.00174 PMC6524469

[51] García-Ayuso D, Di Pierdomenico J, Vidal-Sanz M, Villegas-Pérez MP. Retinal ganglion cell death as a late remodeling effect of photoreceptor degeneration. Int J Mol Sci. 2019;20(18):4649. 31546829 10.3390/ijms20184649 PMC6770703

[52] Adhikari P, Zele AJ, Feigl B. The post-illumination pupil response (PIPR). Invest Ophthalmol Vis Sci. 2015;56(6):3838–3849. 26066752 10.1167/iovs.14-16233

[53] Alexander I, Cuthbertson FM, Ratnarajan G, et al Impact of cataract surgery on sleep in patients receiving either ultraviolet-blocking or blue-filtering intraocular Lens implants. Invest Ophthalmol Vis Sci. 2014;55(8):4999–5004. 24970263 10.1167/iovs.14-14054 PMC4132556

[54] Rukmini AV, Milea D, Aung T, Gooley JJ. Pupillary responses to short-wavelength light are preserved in aging. Sci Rep. 2017;7(1):43832. 28266650 10.1038/srep43832 PMC5339857

[55] Zele AJ, Feigl B, Smith SS, Markwell EL. The circadian response of intrinsically photosensitive retinal ganglion cells. PLoS One. 2011;6(3):e17860. 21423755 10.1371/journal.pone.0017860 PMC3056772

