# Supplementary material for: Recovery in pupillometric non-visual functions following chiasmal decompression in pituitary adenoma
Source: Brain Commun. 2026 Jun 19;8(4):fcag233. doi: 10.1093/braincomms/fcag233 (PMC13403273; doi:10.1093/braincomms/fcag233)
Supplement: fcag233_Supplementary_Data [file fcag233_Supplementary_Data.pdf]

Supplementary Material

Recovery in pupillometric non-visual functions following chiasmal decompression in pituitary adenoma

Daniella Mahfoud[1,2,#], Jensen Ang[3,4,#], Beng-Ti Ang [3,5], Monisha Esther Nongpiur[2,6,7], Dan Milea[2,6,7, 8], Raymond P. Najjar[1,2,7,9*]

1. Eye N' Brain Research Group, Department of Ophthalmology, Yong Loo Lin School of Medicine, National University of Singapore, Singapore
2. Singapore Eye Research Institute, Singapore
3. Department of Neurosurgery, National Neuroscience Institute (Singapore General Hospital campus), Singapore
4. SingHealth Duke-NUS Neuroscience Academic Clinical Programme, Duke-National University of Singapore Medical School, Singapore
5. Neuro-Oncology Research Laboratory, Department of Research, National Neuroscience Institute, Singapore
6. Singapore National Eye Center, Singapore
7. Duke-NUS Medical School, Singapore
8. Department of Ophthalmology, Rothschild Foundation Hospital, 25 rue Manin, Paris, France
9. Department of Biomedical Engineering, College of Design and Engineering, National University of Singapore.

***Corresponding Author**

This supplementary material document includes:

Supplementary Tables:

1. **Supplementary Table 1.** Demographics and clinical characteristics of patients with pituitary adenoma with complete dataset (n=18) and healthy controls (n=41)
2. **Supplementary Table 2.** Demographics and clinical characteristics of patients with pituitary adenoma with visual field recovery (n=5) and healthy controls (n=41)
3. **Supplementary Table 3.** Demographics and clinical characteristics of patients with pituitary adenoma with no visual field recovery (n=13) and healthy controls (n=41)
4. **Supplementary Table 4.** Pupillometric features in patients with pituitary adenoma pre- and post-surgery (n=27) compared to healthy controls (n=41)

Supplementary Figures:

1. **Supplementary Figure 1.** Flowchart of enrolment and exclusion of patients and data
2. **Supplementary Figure 2.** Pupillary light reflex (PLR) parameters pre- and post-surgery (n=27) compared to controls (n=41)
3. **Supplementary Figure 3.** Ophthalmic assessments pre- and post-surgery (n=18) compared to controls (n=41)
4. **Supplementary Figure 4.** Heatmap illustrating the spearman correlation coefficients between all measured variables
5. **Supplementary Figure 5.** Pupillary light response and comparisons of pupillometric parameters in patients without full VFI recovery

Supplementary Results: Individual variability in recovery trajectories

1. **Case 1:** Patient with complete recovery
2. **Case 2:** Patient with partial recovery
3. **Case 3:** Patient with limited recovery

Supplementary Tables

Supplementary Table 1. Demographics and clinical characteristics of patients with pituitary adenoma with complete dataset (n=18) and healthy controls (n=41)

Demographic and clinical characteristics	Controls	Patients with pituitary adenoma		P-value
**N**	41	18		
**Age, median (IQR), years**	53.6 (13.7)	57.4 (22.4)		0.35
**Gender, male no (%)**	19 (46.3)	7 (38.9)		0.60
**Ethnicity**				
**Chinese, no (%)**	40 (97.6)	15 (83.3)		0.08
**Indian, no (%)**	1 (2.4)	1 (5.6)		
**Malay, no (%)**	0	0 (0)		
**Others, no (%)**	0	2 (11.1)		
**Diabetes, no (% with)**	1 (2.4)	1 (5.6)		0.54
**Cataract, no (% with)**	18 (43.9)	12 (66.7)		0.11
**PCIOL, no (% with)**	2 (4.9)	2 (11.1)		0.38
		**Pre-operation**	**Post-operation**	
**Upward displacement of optic Chiasm (UDOC), median (IQR), mm**	-	6.4 (4.1)	0.8 (1.6)	**<0.001#**, -
**Optic chiasm thickness (OpCT), median (IQR), mm**	-	1.1 (0.7)	2.3 (0.8)	**0.001#**, -
**VA, median (IQR), LogMAR**	0.1 (0.2)	0.3 (0.8)	0.2 (0.2)	**0.005#**, **<0.001†**, 0.09‡
**VFMD, median (IQR), dB**	0.0 (2.0)	-6.4 (6.2)	-2.9 (4.1)	**0.003#**, **<0.001†**, **<0.001‡**
**PSD, median (IQR), %**	1.4 (0.7)	7.7 (9.1)	3.3 (4.0)	**0.011#**, **<0.001†**, **<0.001‡**
**VFI, median (IQR), %**	99.0 (2)	83.0 (28.5)	94.5 (9.3)	**0.006#**, **<0.001†**, **<0.001‡**

Mann-Whitney U test was used to compare age, VA, VFMD, PSD, and VFI between controls and pituitary adenoma groups pre- (†) and post-surgery (‡). Wilcoxon signed-rank test was used to compare upward displacement of optic chiasm, optic chiasm thickness, VA, VFMD, PSD, and VFI between patients with pituitary adenoma pre- and post- operation (#). $\chi 2$ test was used to compare all other variables between the two groups. **Abbreviations:** VA, visual acuity; HVF, Humphrey visual field; IQR, interquartile range; LogMAR, logarithm of minimum angle of resolution; PCIOL, posterior chamber intra-ocular lens; PSD, pattern SD; VFI, Visual Field Index; VFMD, visual field mean deviation.

Supplementary Table 2. Demographics and clinical characteristics of patients with pituitary adenoma with visual field recovery (n=5) and healthy controls (n=41)

Demographic and clinical characteristics	Controls	Patients with pituitary adenoma		P-value
**N**	41	5		
**Age, median (IQR), years**	53.6 (13.7)	56.4 (11.4)		0.27
**Gender, male no (%)**	19 (46.3)	1 (20)		0.26
**Ethnicity**				
**Chinese, no (%)**	40 (97.6)	4 (80)		0.07
**Indian, no (%)**	1 (2.4)	1 (20)		
**Malay, no (%)**	0	0 (0)		
**Others, no (%)**	0	0 (0)		
**Diabetes, no (% with)**	1 (2.4)	0 (0)		0.72
**Cataract, no (% with)**	18 (43.9)	5 (100)		0.02*
**PCIOL, no (% with)**	2 (4.9)	0 (0)		0.61
		**Pre-operation**	**Post-operation**	
**Upward displacement of optic Chiasm (UDOC), median (IQR), mm**	-	8.7 (7.9)	0.0 (0.8)	**0.04[#]**, -
**Optic chiasm thickness (OpCT), median (IQR), mm**	-	0.8 (1.1)	2.4 (1.3)	0.08[#], **-**
**VA, median (IQR), LogMAR**	0.1 (0.2)	0.2 (0.7)	0.2 (0.2)	0.27[#], **0.03[†]**, 0.1[‡]
**VFMD, median (IQR), dB**	0.0 (2.0)	-3.7 (7.3)	-0.2 (1.9)	**0.04[#]**, **0.01[†]**, 1.0[‡]
**PSD, median (IQR), %**	1.4 (0.7)	3.8 (9.0)	1.5 (0.4)	0.08[#], **0.01[†]**, 0.9[‡]
**VFI, median (IQR), %**	99.0 (2)	94.0 (25.5)	99.0 (0.5)	0.08[#], **0.02[†]**, 0.9[‡]

Mann-Whitney U test was used to compare age, VA, VFMD, PSD, and VFI between controls and pituitary adenoma groups pre- (†) and post-surgery (‡). Wilcoxon signed-rank test was used to compare Upward displacement of optic Chiasm, Optic Chiasm Thickness, VA, VFMD, PSD, and VFI between patients with pituitary adenoma pre- and post- operation (#). $\chi 2$ test was used to compare all other variables between the two groups. **Abbreviations:** VA, visual acuity; IQR, interquartile range; LogMAR, logarithm of minimum angle of resolution; PCIOL, posterior chamber intra-ocular lens; PSD, pattern SD; VFI, Visual Field Index; VFMD, visual field mean deviation.

Supplementary Table 3. Demographics and clinical characteristics of patients with pituitary adenoma with no visual field recovery (n=13) and healthy controls (n=41)

Demographic and Clinical Characteristics	Controls	Patients with pituitary adenoma		P-value
**N**	41	13		
**Age, median (IQR), years**	53.6 (13.7)	58.4 (29.3)		0.6
**Gender, male no (%)**	19 (46.3)	6 (46.2)		1.0
**Ethnicity**				
**Chinese, no (%)**	40 (97.6)	11 (84.6)		0.1
**Indian, no (%)**	1 (2.4)	0 (0)		
**Malay, no (%)**	0	0 (0)		
**Others, no (%)**	0	2 (15.4)		
**Diabetes, no (% with)**	1 (2.4)	1 (7.7)		0.6
**Cataract, no (% with)**	18 (43.9)	7 (53.8)		0.7
**PCIOL, no (% with)**	2 (4.9)	2 (15.4)		0.3
		**Pre-operation**	**Post-operation**	
**Upward displacement of optic Chiasm (UDOC), median (IQR), mm**	-	6.3 (2.8)	0.8 (2.2)	**0.001#, -**
**Optic chiasm thickness (OpCT), median (IQR), mm**	-	1.3 (0.6)	2.2 (0.8)	**0.006#, -**
**VA, median (IQR), LogMAR**	0.1 (0.2)	0.4 (0.8)	0.2 (0.2)	**0.01#, <0.001†, 0.02‡**
**VFMD, median (IQR), dB**	0.0 (2.0)	-7.4 (10.4)	-3.6 (2.8)	**0.03#, <0.001†, <0.001‡**
**PSD, median (IQR), %**	1.4 (0.7)	9.1 (9.1)	4.6 (3.6)	0.06#, **<0.001†, <0.001‡**
**VFI, median (IQR), %**	99.0 (2)	79.0 (42.5)	91.0 (7.5)	**0.03#, <0.001†, <0.001‡**

Mann-Whitney U test was used to compare age, VA, VFMD, PSD, and VFI between controls and pituitary adenoma groups pre- (†) and post-surgery (‡). Wilcoxon signed-rank test was used to compare Upward displacement of optic Chiasm, Optic Chiasm Thickness, VA, VFMD, PSD, and VFI between patients with pituitary adenoma pre- and post- operation (#). $\chi 2$ test was used to compare all other variables between the two groups. **Abbreviations:** VA, visual acuity; IQR, interquartile range; LogMAR, logarithm of minimum angle of resolution; PCIOL, posterior chamber intra-ocular lens; PSD, pattern SD; VFI, Visual Field Index; VFMD, visual field mean deviation.

Supplementary Table 4. Pupillometric features in patients with pituitary adenoma pre- and post-surgery (n=27) compared to healthy controls (n=41)

Pupillometric feature	Controls	Patients with pituitary adenoma		P-value
		Pre-operation	Post-operation	
**Phasic-Blue, median (IQR), %**	41.1 (12.6)	31.9 (16.9)	36.3 (10.9)	**0.01**[#], **<0.001**[†], 0.13[‡]
**Phasic-Red, median (IQR), %**	37.6 (12.7)	28.3 (19.7)	33.2 (14.7)	**0.02**[#], **<0.001**[†], **0.005**[‡]
**Max-Blue, median (IQR), %**	56.0 (6.8)	42.4 (18.8)	51.6 (12.4)	**0.02**[#], **<0.001**[†], **0.007‡**
**Max-Red, median (IQR), %**	53.3 (9.8)	39.4 (18.9)	49.0 (14.4)	**<0.001**[#], **<0.001**[†], **0.01**[‡]
**PIPR6s, median (IQR), %**	13.6 (7.5)	8.6 (7.8)	13.9 (8.4)	**0.02**[#], **0.001**[†], 0.83[‡]
**PIPR AUC 0-12s, median (IQR), %.s**	202.5 (79.4)	128.6 (106.8)	192.3 (100.1)	**0.03**[#], **<0.001**[†], 0.08[‡]
**PIPR>1.7 slope, median (IQR), %/s**	-1.0 (0.6)	-0.7 (0.5)	-1.2 (0.9)	**0.001**[#], **0.001**[†], 0.9[‡]

Mann-Whitney U test was used to compare pupillometric features between controls and pituitary adenoma groups pre- (†) and post-surgery (‡). Wilcoxon signed-rank test was used to compare these features between patients with pituitary adenoma pre- and post- operation (#). **Abbreviations:** IQR, interquartile range; Phasic-Blue, phasic constriction to blue light; Phasic-Red, phasic constriction to red light; Max-Blue: maximum constriction to blue light; Max-Red: maximum constriction to red light; PIPR6s, post-illumination pupillary response 6 s after light offset; PIPR AUC 0–12s, PIPR area under the curve; PIPR>1.7 slope, redilation slope 1.7s following light offset .

Supplementary Figures

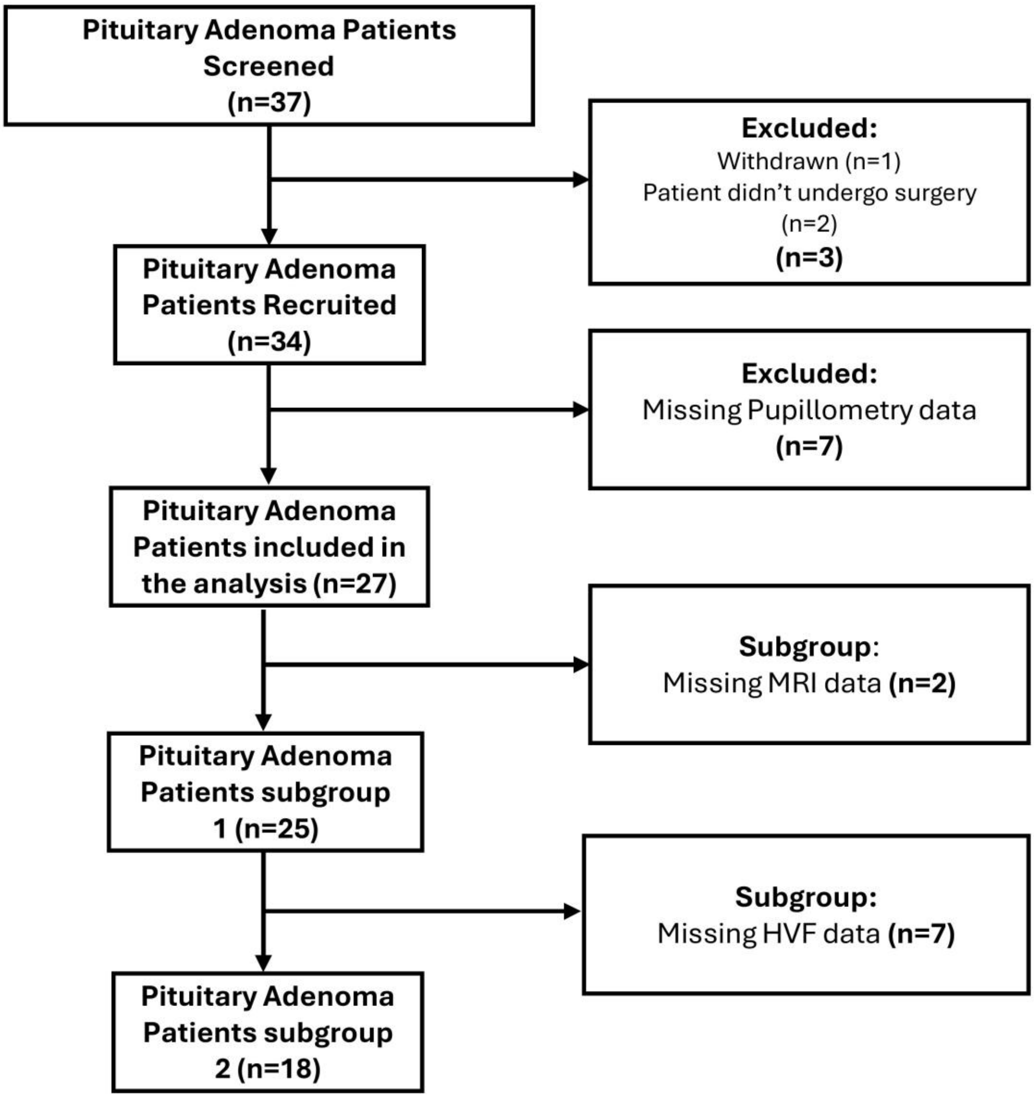

Supplementary Figure 1. Flowchart of enrolment and exclusion of patients and data.
Abbreviations: MRI, Magnetic Resonance Imaging; HVF, Humphrey Visual Field.

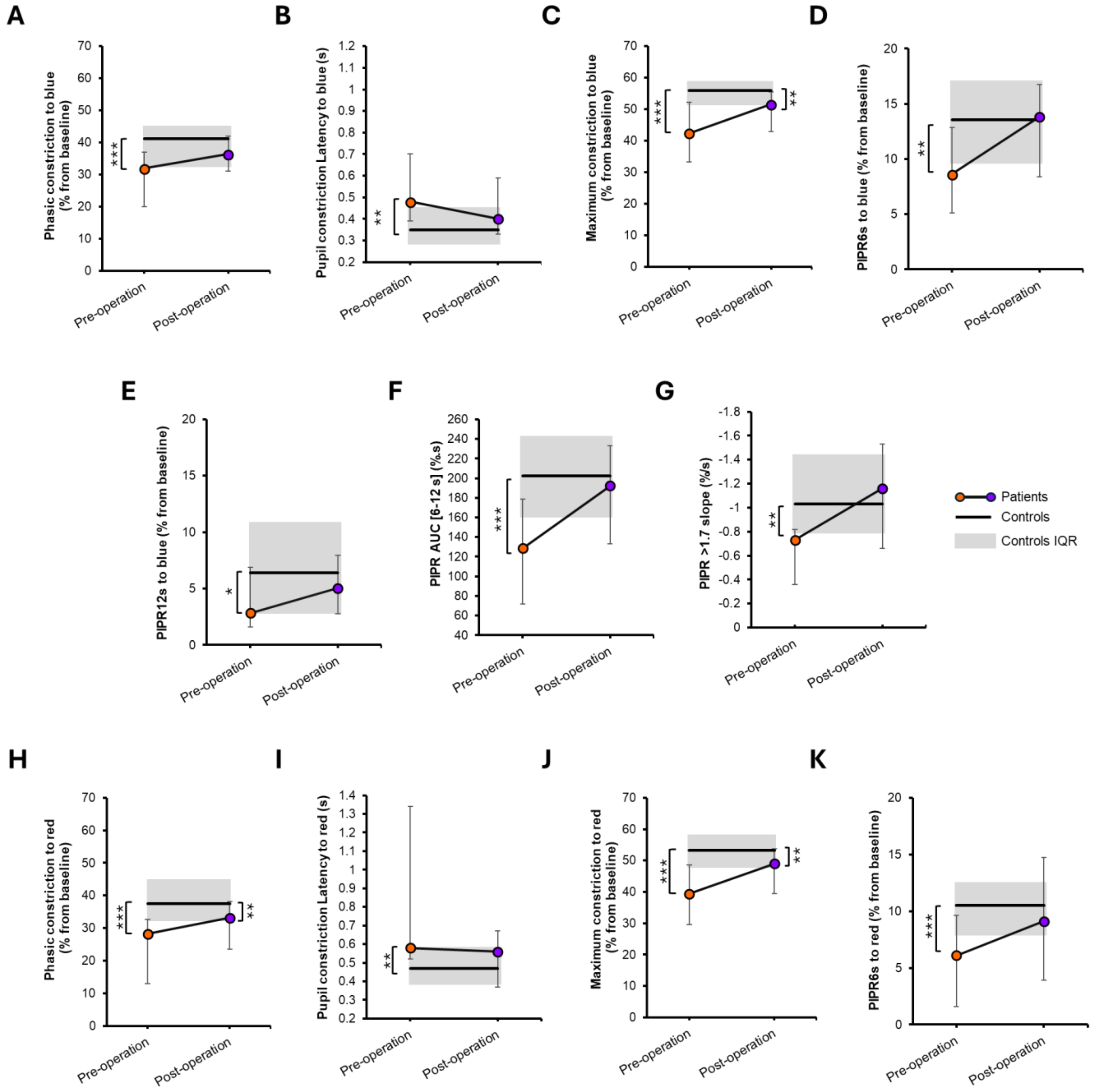

Supplementary Figure 2. Pupillary light reflex (PLR) parameters pre- and post-surgery (n=27) compared to controls (n=41). Each panel illustrates changes in key pupillometric features, with orange and purple markers representing individual pre- and post-operative median values, respectively. The black line represents median values of controls. Gray shaded areas indicate the IQR of control values. Error bars represent IQR within the patient group. Statistical comparisons between groups were performed using a Mann-Whitney U test. *p < 0.05; **p < 0.01; ***p < 0.001. **Abbreviations:** IQR, interquartile range; PIPR, post-illumination pupillary responses.

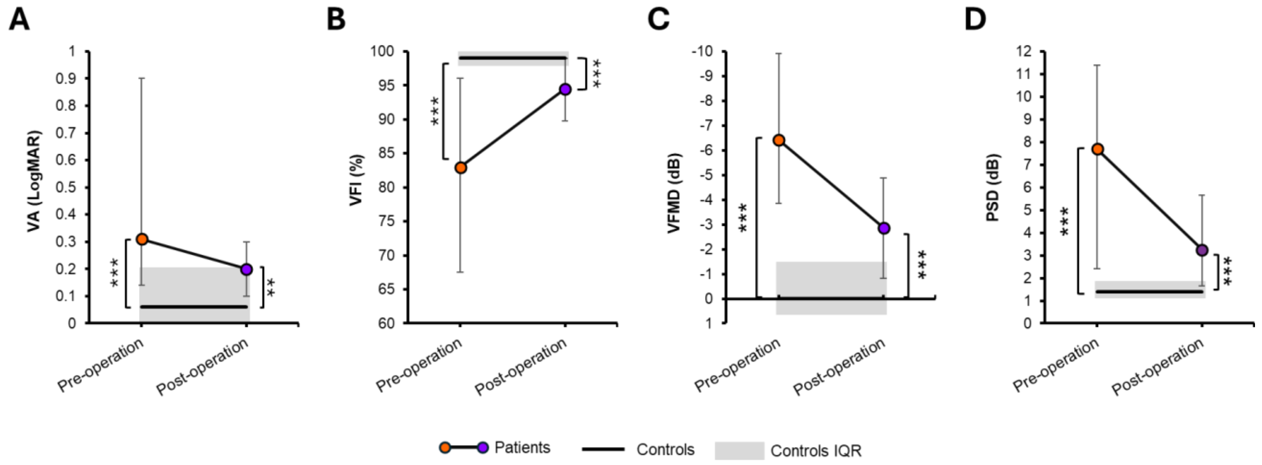

Supplementary Figure 3. Ophthalmic assessments pre- and post-surgery (n=18) compared to controls (n=41). Each panel illustrates changes in ophthalmic outcomes, with orange and purple markers representing individual pre- and post-operative median values, respectively. The black line represents median values of controls. Gray shaded areas indicate the IQR of control values. Error bars represent IQR within the patient group. Statistical comparisons between groups were performed using a Mann-Whitney U test. *$p < 0.05$; **$p < 0.01$; ***$p < 0.001$. **Abbreviations:** IQR, interquartile range; VA, visual acuity; dB, decibels; LogMAR, logarithm of minimum angle of resolution; PSD, pattern SD; VFI, visual field index; VFMD, visual field mean deviation.

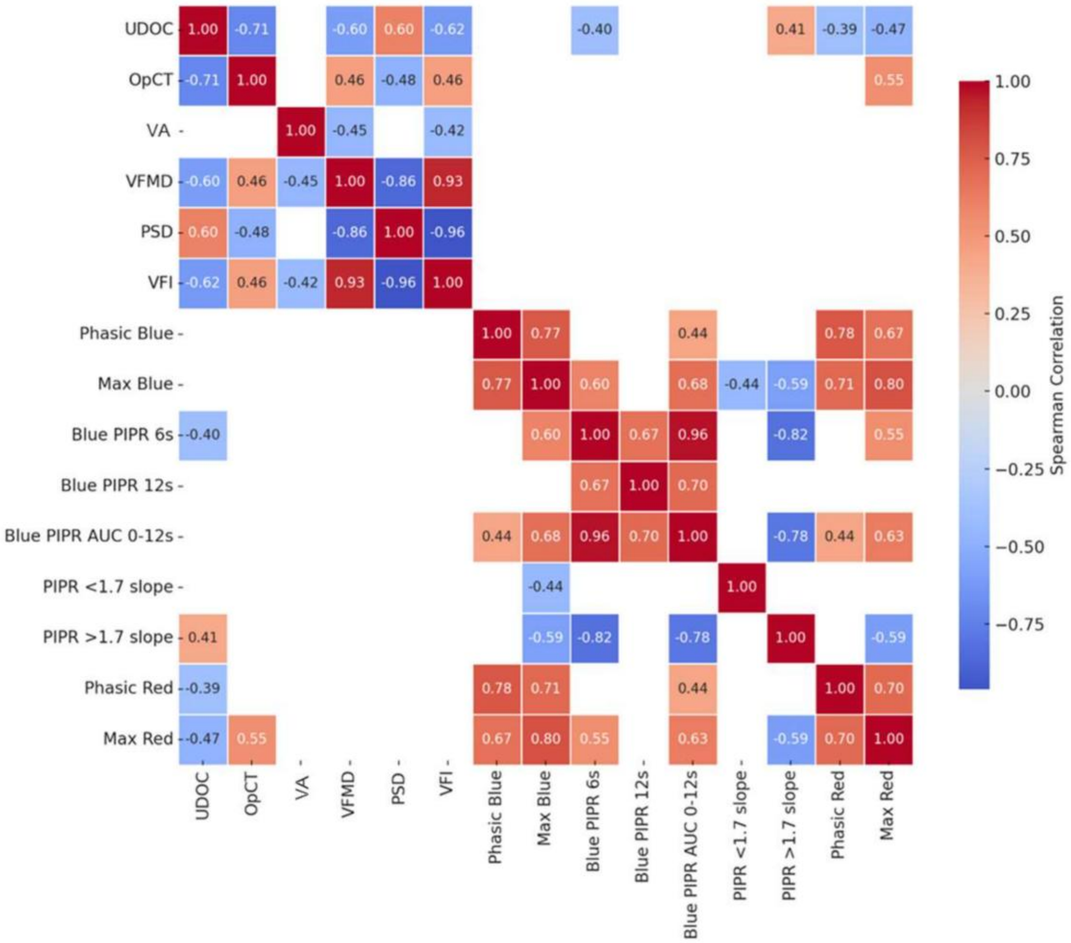

Supplementary Figure 4. Heatmap illustrating the Spearman correlation coefficients between all measured variables after applying the Benjamini-Hochberg (BH) correction for multiple comparisons. Data cover patients having full datasets pre- (n=18) and post- operation (n=18). Only statistically significant correlations (*p*-adjusted < 0.05) are displayed, while non-significant cells are masked to white. Warmer colors indicate positive correlations, whereas cooler colors indicate negative correlations. **Abbreviations:** UDOC, the upward displacement of the optic chiasm; VA, visual acuity; VFI, visual field index; Max Red, maximum constriction to red light; PIPR>1.7 slope, post-illumination pupillary response at >1.7 slope; OpCT, optic chiasm thickness.

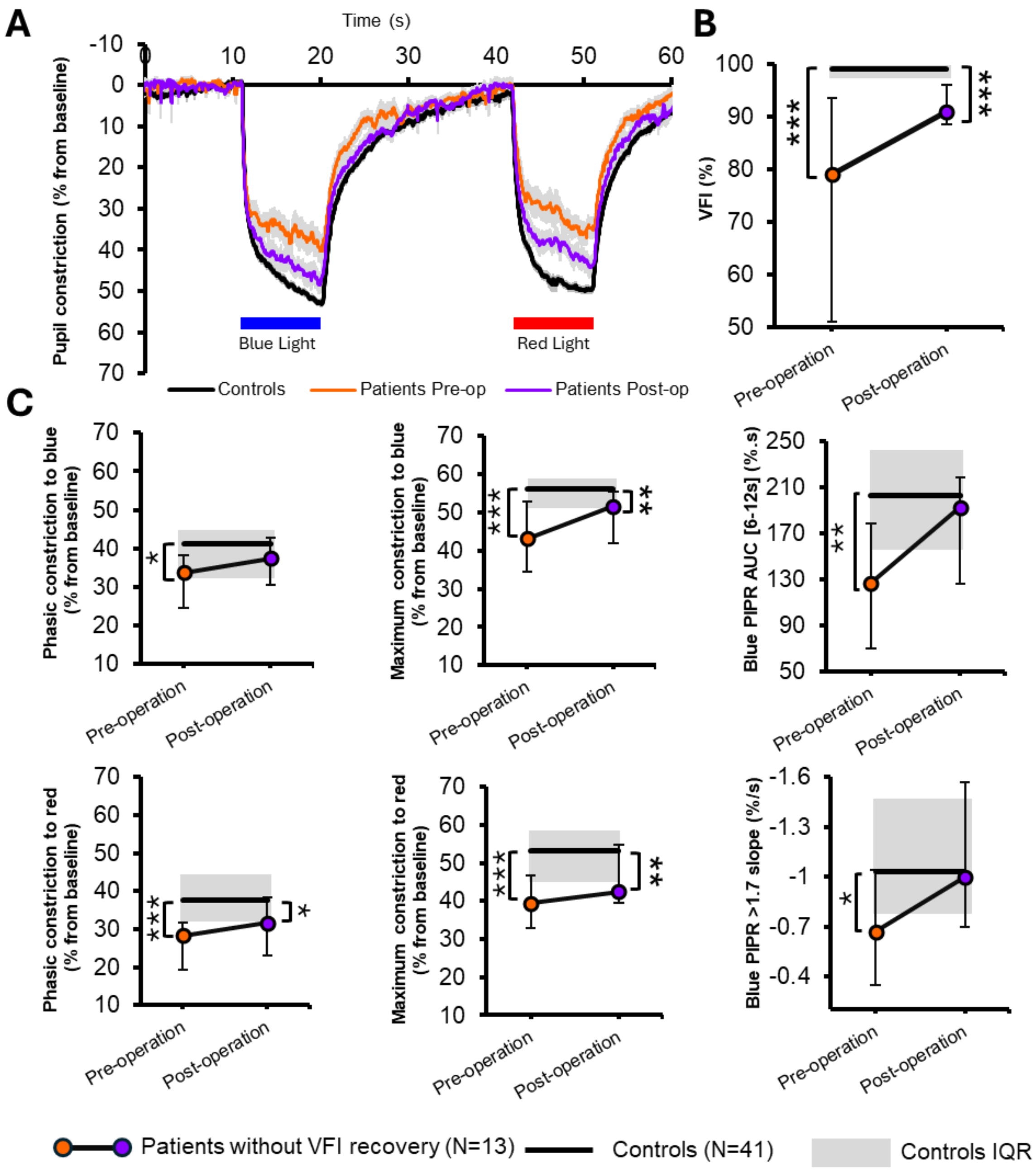

Supplementary Figure 5. Pupillary light response and comparisons of pupillometric parameters in patients without full VFI recovery. **A.** Mean pupil response traces to blue- and red-light stimuli, in patients with pituitary adenoma without VFI recovery (n=13) before (orange) and after surgery (purple) and controls (black). **B.** Difference in VFI in patients with PA before and after surgery. This sub-group of patients does not have a complete visual field recovery. **C.** Differences in main pupillometric features between patients pre- and post-operation and controls. Statistical comparisons between groups were performed using a Mann-Whitney U test. *$p<0.05$; ** $p<0.01$; ***$p<0.001$. **Abbreviations:** IQR, interquartile range; VFI, visual field index; PIPR, post-illumination pupillary response; AUC, area under the curve.

Supplementary Results

Individual variability in recovery trajectories

Individual patient data demonstrated a range of recovery outcomes, with some patients achieving near-normal PLR responses post-operatively, while others exhibited persistent deficits.

Case 1: Patient with complete recovery

Patient 1 presented with a large PA compressing the optic chiasm, as evidenced by pre-operative MRI (9.33 mm displacement) and severe visual field deficits. Pre-operative pupillometry revealed abnormal PLR responses to both blue and red light. Post-surgery, MRI confirmed significant decompression of the optic chiasm (1.57 mm), and visual field testing showed substantial improvement. Pupillometry findings normalized across all features, aligning with improvements in visual field outcomes. No RNFL changes were noticed (RNFL-Pre: 82 μm, RNFL-Post: 81 μm). This case highlights complete recovery across structural (MRI), functional (pupillometry), and visual (HVF) domains, emphasizing the potential for full restoration of visual pathways following TSS in cases with significant decompression (Supplementary Fig. 6).

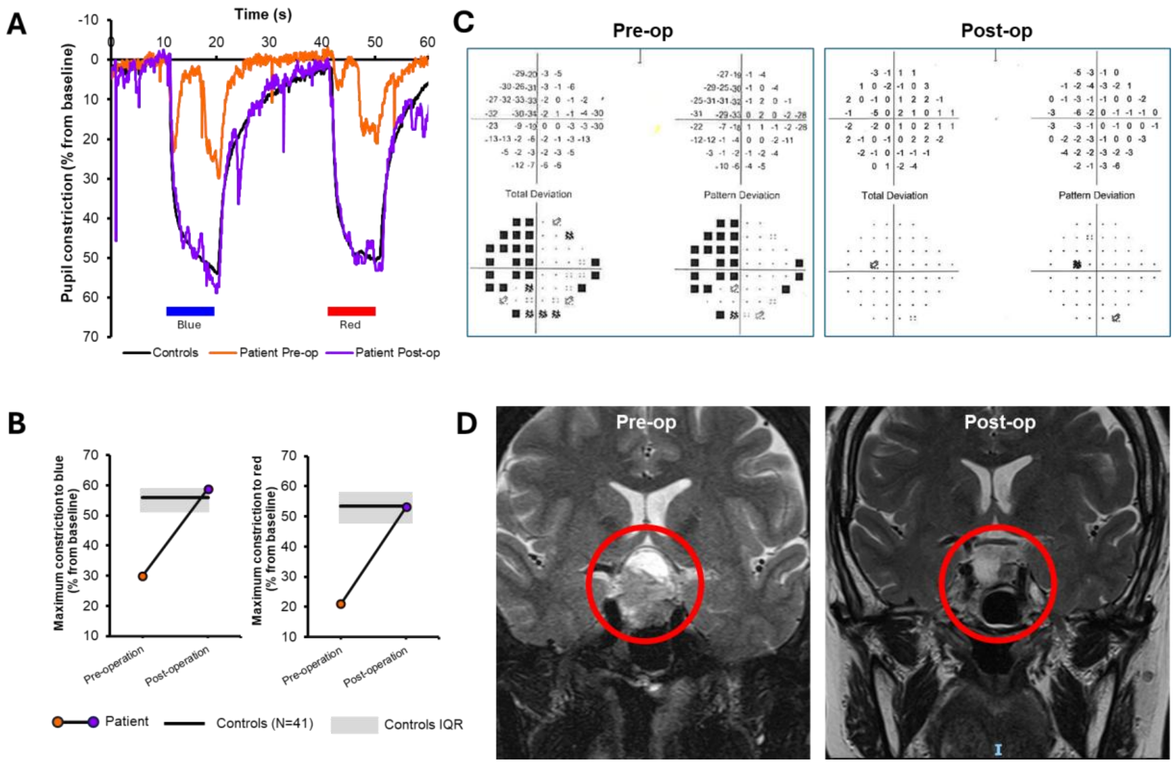

Supplementary Figure 6. Patient 1 multimodal assessment of visual and pupillary function pre- and post-surgery. A. Pupil response traces. **B.** Maximum pupillary constriction to blue and red light pre- and post- operation. **C.** Visual field assessment. **D.** Tumor resection on T2-weighted MRI images before and after surgery.

Case 2: Patient with partial recovery

Patient 2, with a large PA compressing the optic chiasm, presented with pre-operative visual field deficits. Pre-operative MRI revealed 6.6 mm upward displacement of the optic chiasm. Pupillometric assessment showed severe deficits in response to both blue and red-light stimuli. Post-surgical MRI confirmed effective decompression of the optic chiasm (0 mm), and visual field testing showed improvement but not complete recovery. Post-operative pupillometry indicated recovery in pupillary responses to blue light and improvement in red light-mediated responses, though residual deficits persisted in response to red-light in particular. No RNFL recovery was noticed (RNFL-Pre: 85 µm, RNFL-Post: 82 µm). This case demonstrates partial recovery for functional visual domains, with residual deficits in cone-mediated responses indicating the complexity of functional restoration even with effective structural decompression (Supplementary Fig. 7).

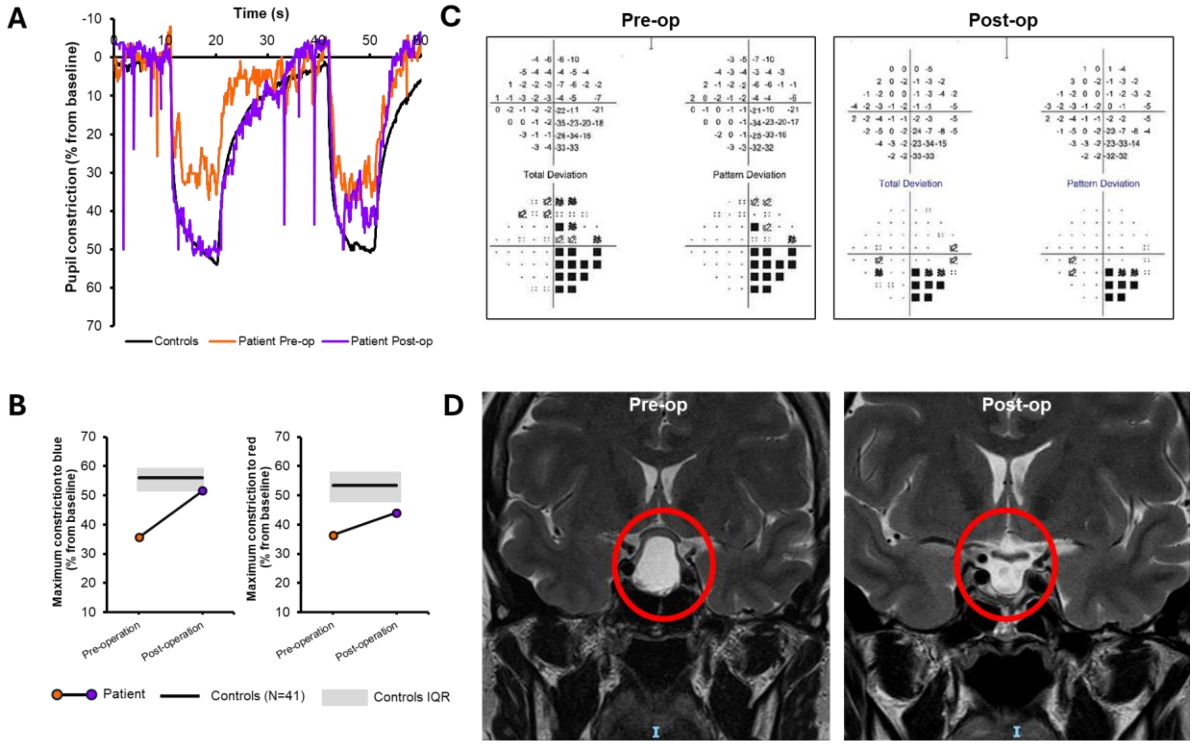

Supplementary Figure 7. Patient 2 multimodal assessment of visual and pupillary function pre- and post-surgery. A. Pupil response traces. **B.** Maximum pupillary constriction to blue and red light pre- and post-operation. **C.** Visual field. **D.** Tumor resection on T2-weighted MRI images before and after surgery.

Case 3: Patient with limited recovery

Patient 3 presented with a PA causing marked optic chiasm compression, as confirmed by pre-operative MRI (4.73 mm displacement). The patient reported visual field loss, with pre-operative visual field testing revealing extensive deficits. Pre-operative pupillometric assessments showed alterations in response to both blue and red-light stimuli. Post-operative MRI demonstrated partial decompression of the optic chiasm (3.46 mm), but residual compression was still evident. Visual field testing showed recovery, with residual defects persisting. Post-operative pupillometry revealed slight improvement in response to blue light; however, red light-mediated responses remained significantly impaired. No RNFL recovery was noticed (RNFL-Pre: 89 μm, RNFL-Post: 86 μm). This case highlights the variability in outcomes and the potential for residual deficits following TSS (Supplementary Fig. 8).

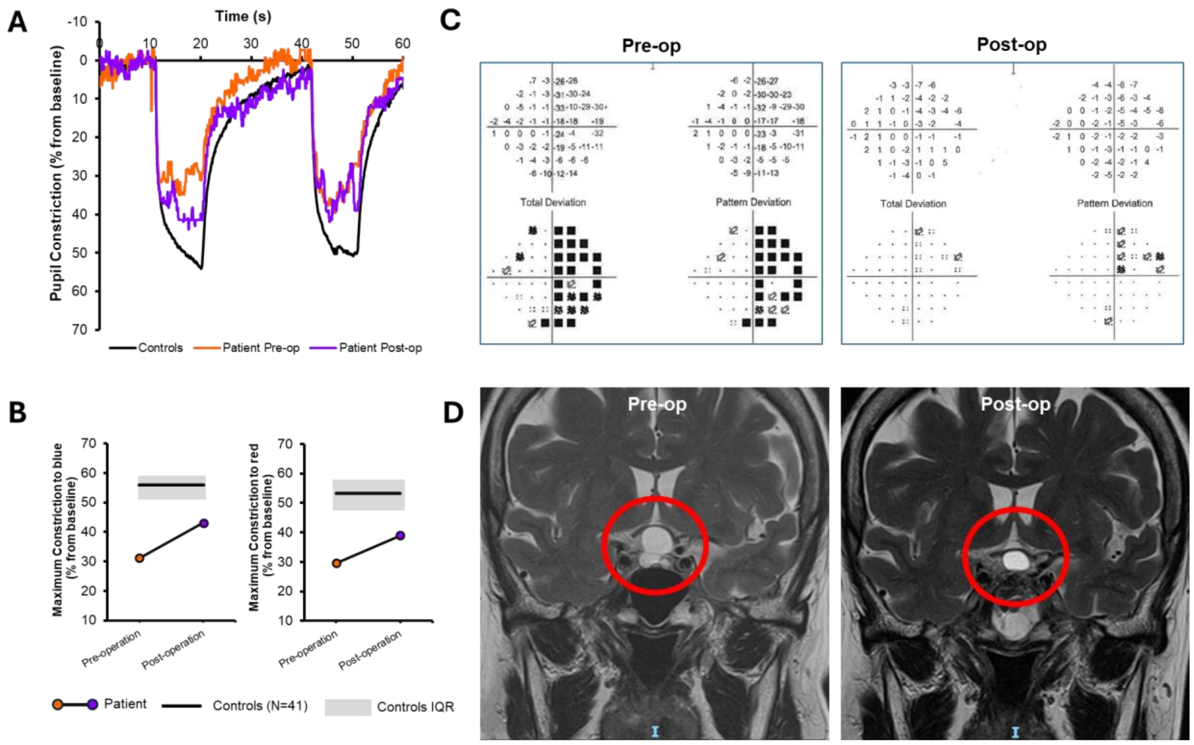

Supplementary Figure 8. Patient 3 multimodal assessment of visual and pupillary function pre- and post-surgery. A. Pupil response traces. **B.** Maximum pupillary constriction to blue and red light pre- and post-operation. **C.** Visual field assessment. **D.** Residual tumor on T2-weighted MRI images before and after surgery.
